# Comparison of Three Serologic Tests for the Detection of Anti-*Coxiella burnetii* Antibodies in Patients with Q Fever

**DOI:** 10.3390/pathogens12070873

**Published:** 2023-06-26

**Authors:** Danilo Alves de França, Mateus de Souza Ribeiro Mioni, Felipe Fornazari, Nássarah Jabur Lot Rodrigues, Lucas Roberto Ferreira Polido, Camila Michele Appolinario, Bruna Letícia Devidé Ribeiro, Ana Íris de Lima Duré, Marcos Vinicius Ferreira Silva, Virgínia Bodelão Richini-Pereira, Helio Langoni, Jane Megid

**Affiliations:** 1 Department of Veterinary Hygiene and Public Health, São Paulo State University, Botucatu 05508-220, Brazil; danilo.franca@unesp.br (D.A.d.F.); mateus.mioni@unesp.br (M.d.S.R.M.); f.fornazari@unesp.br (F.F.); nassarah.lot@unesp.br (N.J.L.R.); camila.appolinario@unesp.br (C.M.A.); bruna.devide@unesp.br (B.L.D.R.); helio.langoni@unesp.br (H.L.); 2 Botucatu Medical School, São Paulo State University, Botucatu 05508-220, Brazil; luxrfp@gmail.com; 3 Ezequiel Dias Foundation, Otávio Magalhães Institute, Belo Horizonte 30510-010, Brazil; ana.dure@funed.mg.gov.br (A.Í.d.L.D.); marcos.silva@funed.mg.gov.br (M.V.F.S.); 4 Regional Laboratories Center II, Adolfo Lutz Institute, Bauru 17015-110, Brazil; virichini@yahoo.com.br

**Keywords:** Q fever, humans, acute disease, serodiagnosis, immunofluorescence, ELISA

## Abstract

The performance of a commercial immunofluorescence assay (IFA commercial), an *in-house* immunofluorescence assay (IFA *in-house*) and an indirect enzyme-linked immunosorbent assay (ELISA) were evaluated in the detection of antibodies anti-*C. burnetii* in the serum of Q fever patients and persons without the disease. For the study, seropositive and seronegative samples for Q fever (*n* = 200) from a serum bank of the Instituto Adolfo Lutz in Brazil were used. Commercial IFA was considered in this study as the gold standard for diagnosing Q fever. The *in-house* IFA demonstrated good agreement with the commercial test, showing high sensitivity (91%) and specificity (97%) compared to the gold standard, with a Kappa coefficient of 0.8954. The indirect ELISA test showed lower agreement with the gold standard, showing low sensitivity (67%), although the specificity of the technique was high (97%) and the Kappa coefficient was moderate (0.6631). *In-house* IFA is an excellent alternative for diagnosing Q fever.

## 1. Introduction

Q fever is an underdiagnosed disease worldwide, mainly in tropical countries of America and Africa, although its occurrence has been identified in several countries in recent years [1]. *Coxiella burnetii* is the causative agent of the disease, a Gram-negative bacterium whose lipopolysaccharide is presented in two phases (I and II) capable of generating antibodies specific for both variants [2]. Higher titers of anti-phase I antibodies in relation to phase II are observed in patients with persistent infections, while higher titers of anti-phase II antibodies are observed in patients with acute disease [3]. Depending on the time of infection, the individual may have both types of circulating antibodies. The presence or absence of these antibodies will determine the type of infection the patient will have and will be useful for serodiagnosis [4].

Serology is a primordial diagnostic method to investigate diseases of lower occurrence or underestimated because it allows broad and rapid testing of the population with a lower cost than that proposed by other diagnostic techniques [5]. However, this type of diagnosis is usually used more often in patients with more than seven days of symptoms, the time required for seroconversion, or in epidemiological investigations [6]. For the diagnosis of Q fever, indirect immunofluorescence (IFA) is the most recommended test because it is more sensitive and specific for the detection of the agent from human samples, while ELISA is the most sensitive and specific test for detection in animal samples [7,8]. Although this is the case, both IFA and ELISA have been used for the diagnosis of Q fever in some countries. Other tests can be used for human diagnoses, such as complement fixation, immunoblotting and radioimmunoassay, although the acquisition of kits for these tests is difficult due to importation [4].

Q fever is most often expressed in its acute form. Acute Q fever reveals nonspecific symptoms, similar to those of a common cold, and may progress either to a natural cure or to a persistent infection. In acute Q fever, phase II immunoglobulin M will initially be produced, more precisely from the second week of symptoms, and remain at high levels until approximately three months after infection. Therefore, this is the best class of immunoglobulins to investigate in patients with suspected acute disease [4]. In the absence of PCR diagnosis and in the impossibility of establishing a paired serum analysis, a single positive convalescent serum sample (stage II ≥ 1:128) in a patient who has been ill for more than seven days indicates probable acute infection [6]. A study by França et al. [3] showed that patients with two and three weeks of symptoms were 2.12 and 2.62 times more likely to be seropositive, respectively, than patients with one week of symptoms. In addition, it was observed that phase II IgM class antibodies were present in 70.5% of the seropositives found in the prevalence study.

Some comparative studies of diagnostic techniques in animal populations have been published; however, no studies evaluating the accuracy of techniques used for human diagnosis have been found [9,10,11,12,13]. In view of this demand, the aim of this paper was to comparatively evaluate the performance of three serological tests used by Q fever diagnostic centers, including an *in-house* test, in detecting antibodies against *C. burnetii* in the serum of people with Q fever and people without the disease.

## 2. Materials and Methods

### 2.1. Study Design

We obtained 200 samples from febrile patients seen by the Brazilian Public Health Service that were stored in a serum bank at the Instituto Adolfo Lutz, São Paulo, Brazil. These sera were used in a Q fever prevalence study [3] and had samples known to be positive. All these sera were submitted to a commercial immunofluorescence assay, considered the gold standard test in this study for the diagnosis of Q fever, revealing a total of 81 seropositive and 119 seronegative samples. Because these were febrile patients with symptoms of acute illness, they were subjected to the specific detection of anti-phase II IgM antibodies, ensuring that they were patients at the onset of infection, suggesting a possible acute Q fever.

### 2.2. Indirect Diagnostic Tests

The analyses were performed at the Rickettsiosis and Hantavirus Laboratory of the Octávio Magalhães Institute, Ezequiel Dias Foundation, Minas Gerais. All 200 serum samples from the study were tested by different serological tests. The specifications of the tests are presented below.

#### 2.2.1. Commercial Indirect Immunofluorescence Assay (Commercial IFA)

A commercial kit (SCIMEDX Corporation, Denville, NJ, USA) containing phase II and phase I antigens from the *C. burnetii* Nine Mile strain (ST-16) was used. This test is capable of detecting anti-phase II IgM antibodies, allowing the characterization of acute infections. Positive and negative controls were from patients previously tested in the laboratory routine.

Initially, aliquots of serum diluted 1:64 in phosphate-buffered saline (PBS; 0.1 M, pH 7.2) were deposited onto slides containing the antigens (30 µL). The slides were incubated (37 °C for 30 min), washed with PBS, and then dried in a humidity chamber. Then, 30 µL of fluorescein isothiocyanate (FITC)-anti-IgM antibody was added to the concavities, followed by another incubation in a moist chamber (37 °C for 30 min), and reading was performed under an immunofluorescence microscope Olympus BX53 (Photonic Solutions Inc., Mississauga, ON, Canada) at 40× objective with the aid of buffered glycerin and coverslip. For each slide used, positive and negative controls of the reaction were prepared in a volume of 30 µL each. The positive samples were serially diluted (1:128, 1:256, 1:512, 1:1024, and so on) until the final titer was reached.

#### 2.2.2. In-House Indirect Immunofluorescence Assay (In-House IFA)

An assay produced *in-house* and used in routine diagnosis by Brazilian public laboratories was used (Ezequiel Dias Fundation, Belo Horizonte, MG, Brazil). This assay contains an antigen originating from *Amblyomma tigrinum* ticks, Argentina strain At12 (ST-73) [14], and produced in embryonated eggs. The particularity of this test compared to the commercial assay is that it is not able to differentiate between the phases of antibodies, and it is not possible to clearly determine whether the patient is an acute or chronic patient. The positive and negative controls were from patients previously tested in the laboratory routine.

Likewise, aliquots of serum diluted 1:64 in phosphate-buffered saline (PBS; 0.1 M, pH 7.2) were deposited on slides containing the antigens (30 µL). The slides were incubated (37 °C for 30 min), washed with PBS, and then dried in a humidity chamber. Then, 30 µL of fluorescein isothiocyanate (FITC)-anti-IgM antibody was added to the concavities, followed by another incubation in a moist chamber (37 °C for 30 min), and reading was performed under an immunofluorescence microscope Olympus BX53 (Photonic Solutions Inc. Ontario, Canada) at 40× objective with the aid of buffered glycerin and coverslip. For each slide used, positive and negative controls of the reaction were prepared in a volume of 30 µL each. The positive samples were serially diluted (1:128, 1:256, 1:512, 1:1024, and so on) until the final titer was reached [15].

#### 2.2.3. Enzyme-Linked Immunosorbent Assay (ELISA)

The SERION ELISA classic *Coxiella burnetii* Phase 2 IgM teste was used. The test was performed according to the manufacturer’s instructions (Institut Virio\Serion GmbH, Würzburg, Germany). The test antigen consists of the Nine Mile strain of *C. burnetii* (ST-16). The samples were previously diluted in a rheumatoid factor solution at a 1:4 dilution and kept for 15 min at room temperature as a kind of pretreatment to prevent false positive reactions with nonspecific IgM antibodies. The samples were then diluted in 1:100 dilution buffer, and 100 µL was applied to the respective wells of the microplates.

The microplates were incubated in a humidity chamber (37 °C for 60 min) and washed 4× with 300 µL. Then, 100 µL of anti-human IgM immunoglobulin conjugate with peroxidase was applied to the wells, and the plates were incubated again in the humidity chamber (37 °C for 30 min). The plates again went through the washing process, and then 100 µL of the substrate solution p-nitrophenylphosphate was applied, which was converted in minutes into the stained product p-nitrophenol. The plates were incubated in a darkroom (37 °C for 30 min). Finally, 100 µL of the stop solution was applied, and the absorbance reading was taken at 405 nm with a reference filter. The positive and negative controls used are provided by the manufacturer.

A quality control certificate is provided in the ELISA Kit showing a table with the calculated OD values and their respective cutoffs. At the beginning of the table, different OD ranges for the standard serum are depicted. According to the average OD of the standard obtained, the corresponding column can be chosen. This column contains the upper and lower OD cutoff values, allowing the evaluation of the patient sample without the need for logistic calculations. OD values below the cutoff were evaluated as negative, and values above the cutoff were evaluated as positive. The implementation of a correction factor is also not necessary in the context of this evaluation table. For each ELISA kit used, there was a specific standard serum and, consequently, a distinct cut-off point. In total, three ELISA kits were used for the analyses, and the cut-off points were 0.250 OD, 0.310 OD and 0.360 OD (OD Range 405 nm, standard serum), serum with values below these considered negative and higher values considered positive. The quality certificate has an intermediate value range, and samples that reach OD in this value range need to be retested in order to confirm seronegativity.

### 2.3. Statistical Analyses

The results of the commercial IFA were compared with the results of the *in-house* IFA and ELISA. The qualitative agreement was evaluated by calculating the Kappa coefficient. Kappa values were interpreted according to the usual scale [16]. Associations between Q fever and serological results were explored by calculating sensitivity, specificity, positive predictive and negative predictive values, accuracy, likelihood ratio of positive test and likelihood ratio of negative test [17]. From a 2 × 2 table (Table 1), the quantitative agreement parameters of the techniques were calculated. For sensitivity and specificity calculations, the formulas (a/a+c) and (d/b+d) were used, respectively. For the calculation of positive predictive values and negative predictive values, the formulas (a/a+b) and (d/c+d) were used, respectively.

## 3. Results

**Figure 1:**
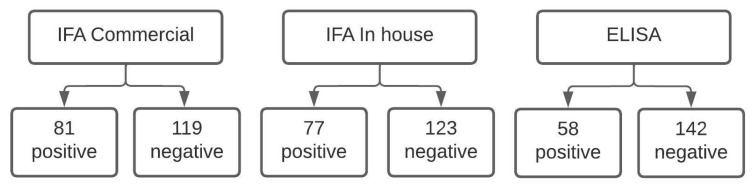
Flowchart of the samples and results of seropositivity.

Figure 1 shows the flow chart of the study, with the positive and negative results of each serological technique.

According to commercial IFA, 40.5% of samples had phase II IgM antibodies, characterizing patients with acute Q fever. In comparison, the *in-house* IFA detected 38.5% of seropositives, and the ELISA detected 29%.

The *in-house* IFA showed high agreement with the commercial test (Cohen’s Kappa 0.8954). When compared to commercial IFA, 91.4% of samples were also positive, while 1.5% of samples were positive by *in-house* IFA alone. Seronegativity was observed in 8.6% of patients with Q fever and 97.4% of patients without Q fever. The results of the associations between *in-house* and commercial IFA can be seen in Table 2.

The sensitivity of the *in-house* test was 91.4% (a/a+c), and the specificity was 97.5% (d/b+d). The positive and negative predictive values were 96.1% (a/a+b) and 94.3% (d/c+d), respectively. According to the Kappa coefficient, the test showed a strong level of agreement (0.8954~0.757–1.034). These and other values are presented in Table 3.

The mean ELISA value was 0.440 OD (95% confidence interval [CI]: 0.360–0.530) for samples from patients with Q fever and 0.140 OD (95% CI: 0.060–0.230) for samples from seronegative patients. The samples whose OD reached intermediate values were retested and kept within the range and were therefore considered seronegative samples, according to the manufacturer’s instructions.

When compared to commercial IFA, 66.6% of samples were also positive, while 2% of samples were positive by ELISA alone. Seronegativation was observed in 33.3% of patients with Q fever and 6.6% of patients without Q fever. The overall ELISA results showed significant differences between the two categories (Table 4).

The sensitivity of the ELISA was 66.7% (a/a+c), and the specificity was 96.6% (d/b+d). Positive predictive and negative predictive values were 93.1% (a/a+b) and 81.0% (d/c+d), respectively. According to the Kappa coefficient, the test showed a moderate level of agreement (0.6631~0.5289–0.7973). These and other values are presented in Table 5.

The proportion of positive samples obtained with *in-house* IFA and ELISA was lower than that obtained with commercial IFA, but the difference was not statistically significant between the techniques. The IFA showed similar results, but in general, the commercial IFA test was slightly more sensitive than the *in-house* IFA. The ELISA showed different sensitivity results than the commercial IFA, with a much lower sensitivity.

The results can be seen in more detail in Table S1.

## 4. Discussion

The *in-house* IFA test has proven to be an excellent alternative for the diagnosis of Q fever and for future research at a reduced cost, presenting very good sensitivity and accuracy values that are very close to the gold standard available on the market. In addition, in Brazil, there are no tests licensed and for sale, so there is greater importance for those countries that are in the same situation. Although the *in-house* test does not specifically detect anti-phase II antibodies, it is likely that the At12 antigen used in the production of the slides has a considerable amount of phase II antigens [2].

It is worth noting that IFA *in-house* has locally produced antigens and conjugates, and this antigen originates from a Latin strain (ST-73) distinct from that used in the sensitization of commercial slides (ST-16). According to Jäger et al. [18] and Beare et al. [19], the genotypes of *C. burnetii* vary greatly depending on the site of origin, which can impact diagnosis. Even so, the results between the techniques were similar, suggesting that the type of *C. burnetii* strain does not have much influence on serological detection, even if they belong to distinct genomic groups, regardless of the country and region in which the tested population originates.

ELISA did not show high sensitivity when compared to the gold standard, and the accuracy was also lower. The lower sensitivity was already expected, according to the recommendation of IFA as the test of choice for human serodiagnosis [4]. Although it was lower, according to the Kappa coefficient, the technique showed a moderate level of agreement, which means that the technique is considered good for epidemiological investigations [16]. On the other hand, the *in-house* technique showed a Kappa coefficient with a strong level of agreement, making it strongly recommended for diagnosis [16]. The use of ELISA is recommended for human diagnosis in the impossibility of obtaining IFA, and in particular, in the diagnosis of the disease in animals.

In addition to the Kappa coefficient, the likelihood ratio of a positive test and the likelihood ratio of a negative test showed significant results and confirmed the two serological techniques evaluated as good diagnostic tests, despite the differences in sensitivity. These values indicated that seropositive people by *in-house* IFA have 36.24 times more chances of having the disease and that seronegative people have only a 0.08865 chance of having the disease. In the case of the ELISA, the values indicated that those who were seropositive had a 19.83 chance of having the disease, and those who were seronegative had a 0.3449 chance of having the disease.

The main application of the *in-house* test would be for epidemiological studies; however, based on the high concordance observed in this study, the test can be recommended for clinical diagnosis, emphasizing that the diagnosis of Q fever should, in addition to serology, be based on the patient’s symptoms and a paired serology to visualize the rise in titers. Other tests can be used in conjunction for diagnosis, as is the case with PCR for the detection of Q fever in patients with a few days of symptoms. The *in-house* test, therefore, is recommended for the clinical diagnosis of Q fever in the absence of a commercial immunofluorescence kit in countries like Brazil, where the importation of kits is difficult. The ELISA test showed moderate agreement and is recommended as a secondary alternative in the absence of any immunofluorescence diagnostic kit. In this case, moderate agreement is seen as acceptable in the diagnosis of Q fever, because the specificity of the test is high, being able to detect most patients without many false positives. Emphasizing again that serology should always be associated with other factors and that patient follow-up is very important. However, we emphasize that IFA is the most sensitive test for the diagnosis of human Q fever and should always be prioritized.

The IgM test provides limited diagnostic value as a stand-alone test. IgM antibodies have lower specificity than IgG, and in the case of *C. burnetii*, they may show cross-reactivity with bacteria of the genera Legionella and Bartonella. However, cross-reacting antibodies usually have low titers and should not result in misdiagnosis [20,21]. This study was limited to patients suggestive of acute disease and in the early phase of the disease; it would be important in future studies to evaluate diagnostic tests for IgG antibody detection and in asymptomatic populations, which constitute the majority of people infected with the bacteria.

This study contributed to the improvement of diagnostic techniques and, consequently, had an impact on Q fever surveillance and control measures. The COVID-19 pandemic has made the medical and health community aware of the importance of studying the available tests for population control and how best to combine them, having equal importance for other important infectious diseases, such as Q fever. Given the lack of studies comparing the different serological tests for the diagnosis of Q fever and the need to invest in *in-house* techniques for a more economical diagnosis, these results could be of great value.

## 5. Conclusions

The *in-house* IFA test proved to be an excellent alternative for the diagnosis of Q fever. The ELISA did not show high sensitivity when compared to the gold standard, and its use as a secondary test is recommended, corroborating the available literature. This study will allow the researchers of Q fever and the health authorities of the countries to understand the particularities of the serological tests available for the diagnosis of the disease. Specifically, for the diagnosis of Q fever in Brazil, we have demonstrated that the *in-house* technique being applied is valid in the absence of licensed kits. The validation of tests in house is very important because it reduces laboratories’ costs in the purchase of diagnostic kits, which is especially essential for developing countries, where importing European kits is often unfeasible. In addition, optimizing a technique in which a local antigen is used, as is the case of At12, can lead to a more accurate diagnosis, especially with regard to the titers achieved in serology and the cutoff defined to consider a patient as having Q fever.

## Figures and Tables

**Table 1:** Standard used for concordance calculations between diagnostic tests.

		Gold Standard		Total
		(+)	(−)	
**Technique to be tested**	**(+)**	a	b	**a+b**
	**(−)**	c	d	**c+d**
Total		**a+c**	**b+d**	a+b+c+d

**Table 2:** Association between the commercial IFA test and *in-house* IFA.

		Commercial IFA		Total
		(+)	(−)	
***in-house* IFA**	**(+)**	74	3	77
	**(−)**	7	116	123
Total		81	119	200

**Table 3:** Results of the comparative analysis between *in-house* IFA and the IFA commercial.

Parameter	Calculation	CI 95% Inferior–Superior	Method
Sensitivity	91.36%	(83.22–95.75)	Wilson’s Points
Specificity	97.48%	(92.85–99.14)	Wilson’s Points
Positive Predictive Value	96.1%	(89.16–98.67)	Wilson’s Points
Negative Predictive Value	94.31%	(88.72–97.22)	Wilson’s Points
Accuracy	95%	(91.04–97.26)	Wilson’s Points
Likelihood Ratio of Positive Test	36.24	(18.81–69.82)	-
Likelihood Ratio of Negative Test	0.08865	(0.06697–0.1174)	-
Cohen’s Kappa	0.8954	(0.757–1.034)	-

Confidence interval: CI.

**Table 4:** Association between the commercial IFA test and ELISA.

		Commercial IFA		Total
		(+)	(−)	
**ELISA**	**(+)**	54	4	58
	**(−)**	27	115	142
Total		81	119	200

**Table 5:** Results of the comparative analysis between ELISA and the IFA commercial.

Parameter	Calculation	CI 95% Inferior–Superior	Method
Sensitivity	66.67%	(55.85–75.97)	Wilson’s Points
Specificity	96.64%	(91.68–98.69)	Wilson’s Points
Positive Predictive Value	93.1%	(83.57–97.29)	Wilson’s Points
Negative Predictive Value	80.99%	(73.75–86.59)	Wilson’s Points
Accuracy	84.5%	(78.84–88.86)	Wilson’s Points
Likelihood Ratio of Positive Test	19.83	(11.93–32.97)	-
Likelihood Ratio of Negative Test	0.3449	(0.3206–0.3711)	-
Cohen’s Kappa	0.6631	(0.5289–0.7973)	-

Confidence interval: CI.

## Data Availability

All relevant data are within the manuscript.

## Supplementary Materials

The following supporting information can be downloaded at: https://www.mdpi.com/article/10.3390/pathogens12070873/s1; Table S1: Results of commercial immunofluorescence (C-IFA), *in-house* immunofluorescence (IH-IFA) and ELISA for the study samples.
