# Supplementary material for: Comparison of Three Serologic Tests for the Detection of Anti-Coxiella burnetii Antibodies in Patients with Q Fever
Source: Pathogens. 2023 Jun 26;12(7):873. doi: 10.3390/pathogens12070873 (PMC10386034; doi:10.3390/pathogens12070873)
Supplement: Supplementary file 1 [file pathogens-12-00873-s001.zip › pathogens-2418404-supplementary.pdf]

Table S1. Results of commercial immunofluorescence (C-IFA), *in-house* immunofluorescence (IH-IFA) and ELISA for the study samples.

Sample	C-IFA	IH-IFA	ELISA	OD	Cutoff ELISA	Titer IFA
S1	1	1	1	0.314	0.250	128
S2	1	1	0	0.115	0.250	64
S3	1	1	1	0.296	0.250	256
S4	1	1	1	0.748	0.250	64
S5	0	0	0	0.125	0.250	NT
S6	1	1	0	0.187	0.250	128
S7	1	1	0	0.132	0.250	128
S8	1	1	0	0.134	0.250	64
S9	1	1	0	0.084	0.250	128
S10	1	1	0	0.084	0.250	128
S11	0	0	0	0.085	0.250	NT
S12	1	0	0	0.125	0.250	128
S13	0	1	0	0.193	0.250	NT
S14	1	1	1	0.680	0.250	1024
S15	1	1	0	0.206	0.250	64
S16	1	1	0	0.188	0.250	128
S17	1	1	0	0.166	0.250	128
S18	1	1	1	0.269	0.250	64
S19	1	0	0	0.140	0.250	128
S20	1	1	0	0.121	0.250	64
S21	1	1	1	0.448	0.250	128
S22	1	1	1	1.341	0.250	128
S23	1	1	1	0.544	0.250	128
S24	1	1	1	0.748	0.250	128
S25	0	0	0	0.040	0.250	NT
S26	1	1	0	0.117	0.250	512
S27	1	0	0	0.057	0.250	128

S28	0	1	1	0.360	0.250	NT
S29	1	1	1	0.845	0.250	128
S30	1	1	1	0.664	0.250	1024
S31	0	0	0	0.161	0.250	NT
S32	0	0	0	0.170	0.250	NT
S33	1	1	1	0.487	0.250	128
S34	1	1	0	0.231	0.250	128
S35	1	1	0	0.234	0.250	128
S36	0	0	0	0.045	0.250	NT
S37	0	0	0	0.081	0.250	NT
S38	0	0	1	0.360	0.250	NT
S39	1	1	0	0.145	0.250	128
S40	0	0	0	0.130	0.250	NT
S41	1	1	1	0.916	0.250	1024
S42	1	1	0	0.088	0.250	128
S43	1	1	0	0.216	0.250	1024
S44	1	1	0	0.107	0.250	128
S45	0	0	0	0.128	0.250	NT
S46	0	0	0	0.111	0.250	NT
S47	0	0	0	0.150	0.250	NT
S48	0	0	0	0.116	0.250	NT
S49	0	0	0	0.062	0.250	NT
S50	0	0	0	0.067	0.250	NT
S51	0	0	0	0.187	0.250	NT
S52	0	0	0	0.109	0.250	NT
S53	0	0	0	0.062	0.250	NT
S54	0	0	0	0.211	0.250	NT
S55	0	0	0	0.101	0.250	NT
S56	0	0	0	0.118	0.250	NT
S57	0	0	0	0.124	0.250	NT

S58	0	0	0	0.086	0.250	NT
S59	1	1	1	2.319	0.250	32768
S60	0	0	0	0.086	0.250	NT
S61	0	0	0	0.242	0.250	NT
S62	0	0	0	0.242	0.250	NT
S63	0	0	0	0.000	0.250	NT
S64	0	0	0	0.075	0.250	NT
S65	0	0	0	0.101	0.250	NT
S66	0	0	0	0.077	0.250	NT
S67	0	0	0	0.029	0.250	NT
S68	0	0	0	0.121	0.250	NT
S69	0	0	0	0.185	0.250	NT
S70	0	0	0	0.148	0.250	NT
S71	0	0	0	0.098	0.250	NT
S72	0	0	0	0.057	0.250	NT
S73	0	0	0	0.179	0.250	NT
S74	0	0	0	0.084	0.250	NT
S75	1	1	1	0.371	0.250	128
S76	0	0	1	0.341	0.250	NT
S77	0	0	0	0.091	0.250	NT
S78	0	0	0	0.199	0.250	NT
S79	0	0	0	0.232	0.250	NT
S80	0	0	0	0.079	0.250	NT
S81	0	0	0	0.150	0.250	NT
S82	0	0	0	0.084	0.250	NT
S83	0	0	0	0.240	0.250	NT
S84	0	0	0	0.087	0.250	NT
S85	0	0	0	0.000	0.250	NT
S86	0	0	0	0.000	0.250	NT
S87	0	0	0	0.000	0.250	NT

S88	0	0	0	0.110	0.250	NT
S89	1	1	1	0.673	0.360	512
S90	1	1	1	0.430	0.360	256
S91	1	1	1	0.535	0.360	1024
S92	1	1	1	0.696	0.360	128
S93	1	1	1	0.679	0.360	256
S94	1	1	1	0.744	0.360	128
S95	1	1	1	0.622	0.360	256
S96	1	1	1	0.438	0.360	64
S97	1	1	1	0.466	0.360	128
S98	1	1	1	0.451	0.360	128
S99	1	1	1	1.377	0.360	1024
S100	1	1	1	0.914	0.360	256
S101	1	1	1	0.448	0.360	64
S102	1	1	1	0.868	0.360	256
S103	1	1	1	0.462	0.360	128
S104	1	0	1	0.625	0.310	128
S105	1	1	1	0.586	0.310	128
S106	1	1	1	0.599	0.310	256
S107	1	1	1	0.468	0.310	128
S108	1	1	1	0.446	0.310	128
S109	0	1	1	0.385	0.310	NT
S110	1	1	1	0.549	0.310	128
S111	1	1	1	0.327	0.310	64
S112	1	0	1	0.341	0.310	64
S113	1	1	1	0.369	0.310	128
S114	1	1	1	0.384	0.310	64
S115	1	1	1	0.400	0.310	64
S116	1	1	1	0.458	0.310	128
S117	1	1	1	0.371	0.310	64

S118	1	1	1	0.427	0.310	64
S119	1	1	1	0.429	0.310	128
S120	1	1	1	0.322	0.310	64
S121	1	1	1	0.432	0.310	64
S122	1	1	1	0.324	0.310	64
S123	1	1	1	0.406	0.310	128
S124	1	1	1	0.320	0.310	64
S125	1	1	1	0.434	0.310	128
S126	1	1	1	0.456	0.310	128
S127	1	1	1	0.464	0.310	128
S128	1	1	1	0.435	0.310	128
S129	0	0	0	0.122	0.310	NT
S130	1	1	0	0.212	0.310	64
S131	1	1	0	0.124	0.310	64
S132	0	0	0	0.185	0.310	NT
S133	1	1	0	0.236	0.310	64
S134	0	0	0	0.066	0.310	NT
S135	0	0	0	0.117	0.310	NT
S136	0	0	0	0.149	0.310	NT
S137	0	0	0	0.252	0.310	NT
S138	0	0	0	0.079	0.310	NT
S139	0	0	0	0.092	0.310	NT
S140	0	0	0	0.190	0.360	NT
S141	0	0	0	0.191	0.360	NT
S142	0	0	0	0.125	0.360	NT
S143	0	0	0	0.187	0.360	NT
S144	0	0	0	0.115	0.360	NT
S145	0	0	0	0.140	0.360	NT
S146	0	0	0	0.065	0.360	NT
S147	0	0	0	0.146	0.360	NT

S148	0	0	0	0.155	0.360	NT
S149	0	0	0	0.060	0.360	NT
S150	0	0	0	0.274	0.360	NT
S151	0	0	0	0.161	0.360	NT
S152	0	0	0	0.176	0.360	NT
S153	0	0	0	0.140	0.360	NT
S154	0	0	0	0.090	0.360	NT
S155	0	0	0	0.156	0.360	NT
S156	0	0	0	0.238	0.360	NT
S157	0	0	0	0.142	0.360	NT
S158	0	0	0	0.134	0.360	NT
S159	0	0	0	0.204	0.360	NT
S160	0	0	0	0.068	0.360	NT
S161	0	0	0	0.226	0.360	NT
S162	0	0	0	0.165	0.360	NT
S163	0	0	0	0.122	0.360	NT
S164	0	0	0	0.063	0.360	NT
S165	0	0	0	0.108	0.360	NT
S166	0	0	0	0.191	0.360	NT
S167	0	0	0	0.066	0.360	NT
S168	0	0	0	0.154	0.360	NT
S169	0	0	0	0.253	0.360	NT
S170	0	0	0	0.324	0.360	NT
S171	0	0	0	0.134	0.360	NT
S172	0	0	0	0.054	0.360	NT
S173	0	0	0	0.208	0.360	NT
S174	0	0	0	0.088	0.360	NT
S175	0	0	0	0.228	0.360	NT
S176	1	1	0	0.150	0.360	64
S177	1	1	0	0.287	0.360	128

S178	0	0	0	0.246	0.310	NT
S179	0	0	0	0.084	0.310	NT
S180	0	0	0	0.127	0.310	NT
S181	0	0	0	0.249	0.310	NT
S182	0	0	0	0.092	0.310	NT
S183	0	0	0	0.307	0.310	NT
S184	0	0	0	0.144	0.310	NT
S185	0	0	0	0.113	0.310	NT
S186	0	0	0	0.205	0.310	NT
S187	0	0	0	0.125	0.310	NT
S188	1	0	0	0.130	0.360	64
S189	1	0	0	0.161	0.360	64
S190	0	0	0	0.119	0.360	NT
S191	0	0	0	0.156	0.360	NT
S192	0	0	0	0.095	0.360	NT
S193	0	0	0	0.085	0.360	NT
S194	0	0	0	0.285	0.360	NT
S195	0	0	0	0.126	0.360	NT
S196	0	0	0	0.157	0.360	NT
S197	0	0	0	0.104	0.310	NT
S198	0	0	0	0.271	0.310	NT
S199	0	0	0	0.098	0.310	NT
S200	0	0	0	0.102	0.310	NT

1: positive; 0: negative. NT: no titer.
